# Cbl-b silenced human NK cells respond stronger to cytokine stimulation

**DOI:** 10.1186/2051-1426-3-S2-P230

**Published:** 2015-11-04

**Authors:** Guenther Lametschwandtner, Monika Sachet, Isabella Haslinger, Hannes Mühleisen, Hans Loibner

**Affiliations:** 1 Apeiron Biologics, Vienna, Austria; 2 Department of Surgery, Medical University Vienna, Vienna, Austria

## 

The E3 ubiquitin ligase cbl-b has been identified as an important gatekeeper limiting T cell activation and more recently also NK cell activation. Hence, cbl-b deficient NK cells displayed stronger anti-tumor responses and this has been linked to a key role of cbl-b for modulation of TAM-receptors. However, in T cells cbl-b has been described to interact with different key regulators of T cell receptor and costimulatory signaling pathways. We have therefore investigated, whether abrogation of cbl-b function in human NK cells mediates increased responsiveness to cytokine stimulation.

Primary human NK cells were isolated and cbl-b silenced by electroporation with siRNA directed against cbl-b. Cbl-b silenced NK cells reacted stronger to tumor cell contact and this response was synergistically enhanced by cytokine stimulation with IL-2 and IL-12. Moreover, stimulation of cbl-b silenced NK cells with cytokines in the absence of tumor cells, either with IL-2 and IL-12 or IL-2 alone led to enhanced activation of NK cells, resulting in increased secretion of effector cytokines like IFN-g and TNF-a and upregulation of the activation marker CD69. Similar results were obtained for stimulation of cbl-b silenced T cells with other key cytokines of innate immune responses, particularly type I Interferons like IFN-a and IFN-b. These data demonstrate that the proposed central role of cbl-b as a negative regulator of various signaling pathways, including Akt, also applies to NK cells.

Together, these results show that interfering with cbl-b function in human NK cells enables synergistic responses to key cytokines of innate and adaptive immune responses. Thus, targeting cbl-b in the context of tumor-immune therapy should not be confined to the T cell compartment, but include NK cells as well. This can be achieved in the context of adoptive cell therapies, when autologous patient PBMCs are silenced for cbl-b ex vivo and retransferred afterwards. Such a protocol was established and is currently tested in a Phase I trial at Wake Forest University. The observed synergism of cbl-b silencing and NK cell stimulation by cytokines like IL-2 could be of particular relevance when tumor-reacting cbl-b silenced T cells infiltrate the tumor and secrete enhanced amounts of IL-2. In addition, it also provides a rationale for combinations of cbl-b targeting approaches with local application of cytokines at the tumor site (e.g. intratumoral injection of IL-2).

